# MEMS Tunable Metasurfaces Based on Gap Plasmon or Fabry–Pérot Resonances

**DOI:** 10.1021/acs.nanolett.2c01692

**Published:** 2022-08-18

**Authors:** Paul C. V. Thrane, Chao Meng, Fei Ding, Sergey I. Bozhevolnyi

**Affiliations:** † Centre for Nano Optics, University of Southern Denmark, Campusvej 55, Odense DK-5230, Denmark; ‡ SINTEF Smart Sensors and Microsystems, Gaustadalleen 23C, 0737 Oslo, Norway

**Keywords:** Metasurface, Tunable, MEMS, Gap Surface Plasmon, Plasmonic, Fabry−Pérot, Intercell Coupling

## Abstract

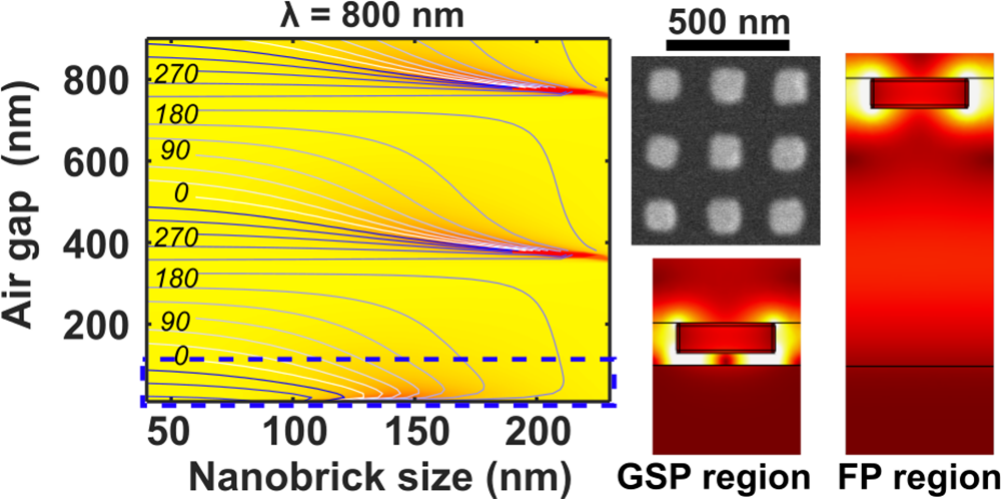

Tunable metasurfaces promise to enable adaptive optical systems with complex functionalities. Among possible realizations, a recent platform combining microelectromechanical systems (MEMS) with gap-surface plasmon (GSP) metasurfaces offers high modulation efficiency, broadband operation, and fast response. We compare tunable metasurfaces operating in GSP and Fabry–Pérot (FP) regions by investigating polarization-independent blazed gratings both numerically and experimentally. Peak efficiency is calculated to be ∼75% in both cases (∼40% in measurements), while the operation bandwidth is found larger when operating in the GSP region. Advantages of operating in the FP region include relaxed assembly requirements and operation tolerances. Additionally, simulation and experimental results show that coupling between neighboring unit cells increases for larger air gaps, resulting in deteriorated efficiency. We believe the presented analysis provides important guidelines for designing tunable metasurfaces for diverse applications in miniaturized adaptive optical systems.

## Introduction

Metasurfaces have successfully demonstrated a wide range of optical effects and components,^1−5^ with a lot of recent research focusing on developing metasurfaces with tunable properties to enable adaptive optical components and with several different techniques being followed, each having their own advantages and disadvantages.^6−9^ One method to achieve this tunability is, for instance, to include materials that undergo a phase change. GeSbTe can, for example, change from having a crystal structure to an amorphous state depending on the temperature, with the two states having very different permittivity.^10^ By incorporating resistive heaters, it is thus possible to make metasurface elements that can change their resonances quite significantly with a drawback being that demonstrated devices have slow switching time.^11^ Faster responses have been demonstrated using the electro-optic effect in lithium niobate^12,13^ or by modulating the free carrier density using electric^14^ or optical^15^ signals, with an issue being that the permittivity changes are limited to thin accumulation or depletion layers giving low modulation ranges.^7^ The effect can be enhanced by using ε-near-zero materials^16^ or by using 2D materials such as graphene^17^ or black phosphorus.^18^ Liquid crystals enable larger and more efficient modulation by changing the refractive index around the nanostructures^7^ but again have slower responses due to the time it takes to rotate the molecules.^19^ Metasurfaces can also be adjusted through mechanically altering the system, with demonstrated concepts including embedding the nanostructures in a stretchable polymer^20^ or incorporating the metasurface with MEMS.^21^ MEMS based tunable metasurfaces can achieve high efficiency modulation while still switching fast enough for many applications depending on the specific mechanical implementation, with most systems being able to operate in the range from one kHz up to several hundred kHz.^22^ For visible and near-IR frequencies the individual meta-atoms are so small that individual actuation by MEMS is challenging, while collective modulation of all meta-atoms is more straightforward.

One such recently demonstrated platform^23^ consists of a gold MEMS mirror^24^ and a glass substrate with gold nanostructures, where the air gap between the nanostructures and mirror can be controlled accurately. The system is designed to function as a reflective optical metasurface (OMS) for light with wavelength λ = 800 nm when the air gap is less than 50 nm. For these small separations there are GSP resonances^25^ due to the near field coupling of the nanostructures and mirror. By moving the mirror away, these GSP resonances disappear, switching off the metasurface functionality and replacing it with that of a standard mirror. The experimentally demonstrated efficiency of this system was 50%, with switching times less than 0.4 ms. In this work we describe how the same MEMS-OMS platform can function also for larger air gaps owing to hybrid plasmonic FP resonances.^26,27^ This configuration has recently been used to achieve efficient and fast 0–2π birefringence control in reflection.^28^ Not only is fabrication easier at larger air gaps since any particle or unevenness may obstruct the MEMS mirror from getting close enough for the GSP resonances, but larger gaps could also help reduce the trade-off between aperture size and switching speed by alleviating squeeze film air damping in the system.^29^ Additionally, the amount of simulations required for design is reduced through the use of the analytic FP equation, removing the need to simulate the response for every air gap separately. We show also that the simulated peak efficiency is around 75% for metasurfaces working in both GSP and FP regions, while the bandwidth is larger for the GSP metasurface with around 2 times the bandwidth when comparing with the metasurface working at the first FP resonance. For larger air gaps there is progressively more coupling/cross-talk between neighboring nanostructures due to scattering and multiple reflections in the FP cavity, resulting in a gradual decrease in metasurface efficiency. This is a result of the metasurface design being based on simulations where the unit cells are placed in arrays of identical structures, whereas the actual metasurface may generally consist of varying meta-atoms. We verify this effect both numerically and experimentally.

## Results and Discussion

**Figure 1:**
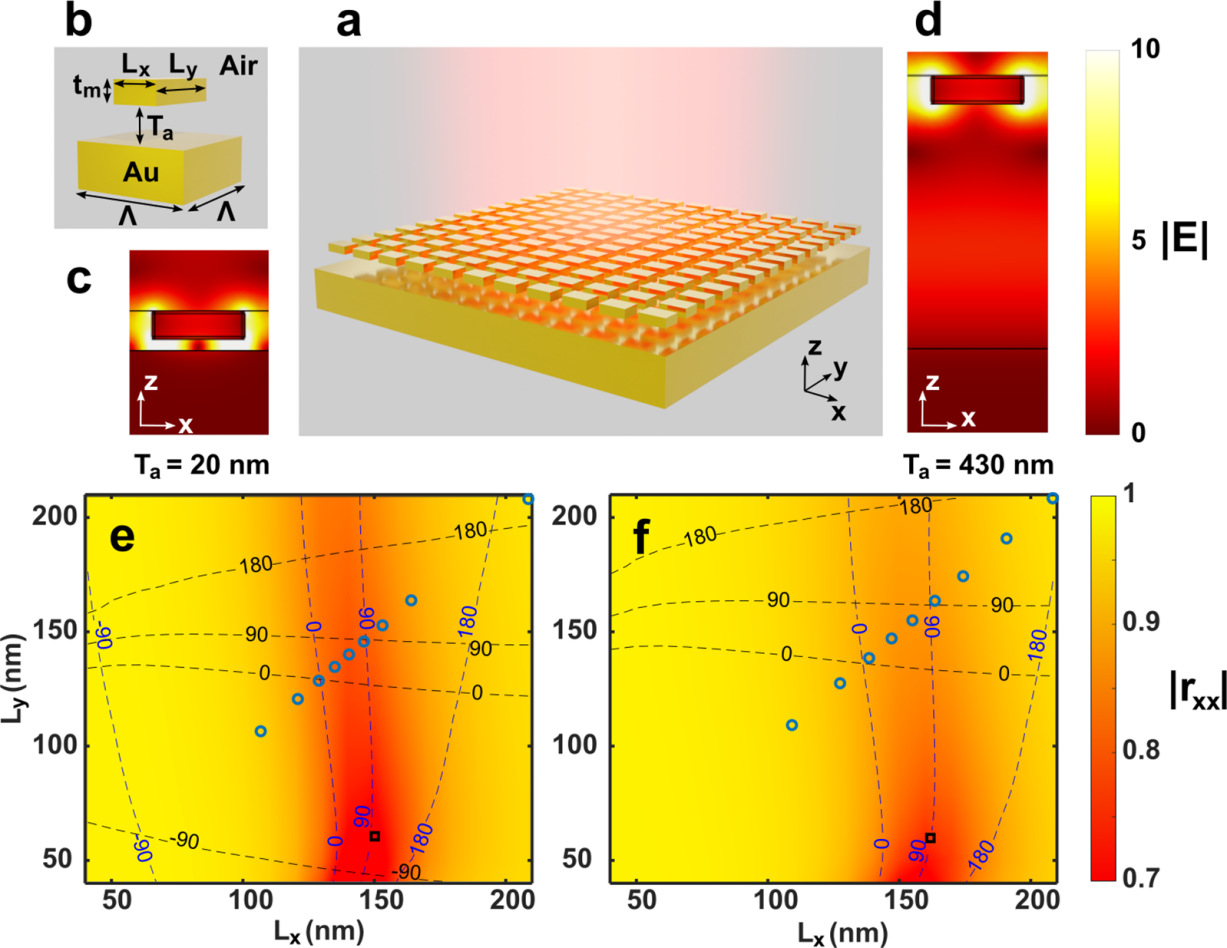
MEMS-OMS unit cell design within respective GSP and FP regions. (a, b) Schematic illustration of the MEMS-OMS metasurface and unit cell. A gold brick with side lengths *L*_*x*_ and *L*_*y*_ and thickness *t*_*m*_ of 50 nm is situated a distance *T*_a_ away from a gold substrate. The unit cell has a square footprint with side lengths Λ = 250 nm. (c, d) The norm of the electric field in the *xz* plane at the center of the nanobricks for *x*-polarized excitation at normal incidence with separation distances of *T*_a_ = 20 nm and *T*_a_ = 430 nm, respectively. The nanobrick geometries are indicated as black squares in (e) and (f). (e, f) Absolute value of the complex reflection coefficients calculated as a function of nanobrick dimensions *L*_*x*_ and *L*_*y*_ at the wavelength of λ = 800 nm for (e) *T*_a_ = 20 nm and (f) *T*_a_ = 430 nm. The color maps represent the reflection amplitude for *x*-polarized excitation at normal incidence, while the blue and black contour lines indicate the reflection phases acquired for *x*- and *y*-polarized excitations, respectively. Blue circles indicate nanobrick geometries for composing the polarization independent MEMS-OMS blazed gratings optimized in respective GSP and FP regions. The phase and reflection amplitudes of these nanobricks are shown in Figure S1.

To compare plasmonic metasurfaces designed to work in the GSP and FP regions, we first calculate the complex reflection coefficient for different nanostructure geometries at two nanostructure–substrate separations corresponding to the two regions. Figure 1a and Figure 1b illustrate the MEMS-OMS and its constituent unit cells used in this work. Specifically, the periodically repeated unit cell has a side length Λ and consists of a gold nanobrick with thickness *t*_*m*_ and side lengths *L*_*x*_ and *L*_*y*_ and separated from a gold substrate by an air gap *T*_a_. In physical implementations there needs to be a dielectric substrate supporting the nanobricks; this has been omitted in the simulations except when comparing with the experimental measurements discussed later. To design the dynamic MEMS-OMS, we set the working wavelength at λ = 800 nm and choose the unit cell size of 250 nm to avoid any high-order diffraction and excitation of surface waves. Meanwhile, the optimal nanobrick thickness *t*_*m*_ is found to be 50 nm, ensuring large reflection amplitudes and wide phase coverage.^23,30^Figure 1c and Figure 1d show electric field plots for two configurations of the unit cell, while Figure 1e and Figure 1f show the reflection coefficients as a function of *L*_*x*_ and *L*_*y*_ for two different values of *T*_a_ for normally incident *x*-polarized excitation at λ = 800 nm. At *T*_a_ = 20 nm, there is a GSP resonance around *L*_*x*_ = 150 nm with near field coupling between the substrate and nanobrick as can be seen in the plot of the electric field in Figure 1c. At *T*_a_ = 430 nm, there is a less sharp resonance centered around *L*_*x*_ = 160 nm, due to a hybrid plasmonic/FP resonance between the substrate and the layer of nanobricks since the near-field coupling between the nanobricks and substrate is negligible, as shown in Figure 1d. As can be seen by comparing the phase contours in Figure 1e and Figure 1f, the range of available reflection phases are slightly larger for the case where *T*_a_ = 20 nm; however the difference is not significant enough to give larger efficiencies for the blazed grating designs presented later. The phase profiles for these gratings are shown in Figure S1.

**Figure 2:**
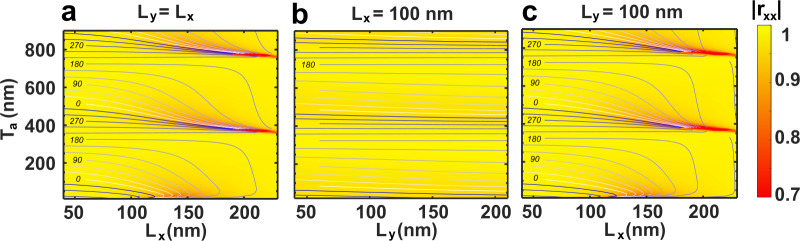
Complex reflection coefficients as a function of air gap and nanobrick dimensions. The color map represents the reflection amplitudes for normally incident *x*-polarized light, while the contour lines indicate the reflection phases. The three subfigures represent three different cases of the nanobrick dimensions, namely, (a) square bricks (*L*_*x*_ = *L*_*y*_), (b) constant *L*_*x*_ = 100 nm, and (c) constant *L*_*y*_ = 100 nm. The light is normally incident and with wavelength 800 nm.

The transition between these GSP and FP regions is displayed in Figure 2, where the reflection coefficients for different brick sizes are shown as a function of *T*_a_. The first FP resonance is located around *T*_a_ = 350 nm with *T*_a_ + *T*_*m*_/2 close to λ/2, with a difference corresponding to the phase change upon reflection on the gold mirror. At this separation the system acts as a gold mirror with the reflection coefficient being independent of *L*_*x*_ and *L*_*y*_, since at this separation distance the nanostructures are centered in the interference minimum from the superposition of incident and relected fields. For slightly larger air gaps the reflection is very dependent on the nanobrick dimensions (e.g., *T*_a_ = 430 nm as shown in Figure 1f) and then gradually returns to mirror-like behavior at the second FP resonance located around *T*_a_ + *T*_*m*_/2 close to λ, with the pattern repeating for subsequent FP apart by a spaced separation of λ/2, again with a small correction due to the phase change upon multiple reflections on the gold mirror. This can be accurately described with the FP equation:^26^
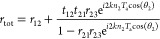
1where the total reflection coefficient *r*_tot_ is given as a function of the reflection and transmission coefficients *r*_*ij*_ and *t*_*ij*_, with the light incident on region *j* from region *i* and the subscripts 1, 2, and 3 respectively referring to the regions above the nanobricks, between the nanobricks and substrate, and the substrate. Note that the reflection and transmission coefficients in general are dependent on the polarization and incidence angle of the light. With mirror-symmetric nanostructures and both regions 1 and 2 consisting of the same material (e.g., air), we have *r*_12_ = *r*_21_, *t*_12_ = *t*_21_, and *n*_2_ = 1. *T*_a_ cos(θ_2_) is the effective air gap for light traversing the gap with an angle θ_2_ and can be simplified as *T*_a_ for normal incidence. By simulating the structures without any gold substrate, the reflection and transmission coefficients *r*_*ij*_ and *t*_*ij*_ are determined for each nanobrick geometry. Equation 1 is then used to calculate the total reflection coefficient as a function of *T*_a_, thus avoiding the requirement of simulating the full structure for every air gap separation. This method gives correct results except for very small air gaps, in this case *T*_a_ < 80 nm = λ/10, where near-field coupling and corresponding GSP excitation must be taken into account. The difference between the results from eq 1 and full-wave simulations including the substrate can be clearly observed for small gap sizes in Figure S2.

**Figure 3:**
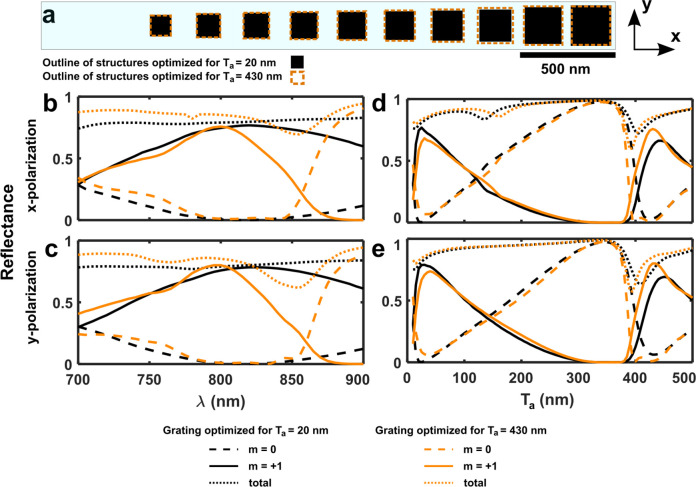
Dynamic MEMS-OMS blazed gratings designed for respective GSP and FP regions. (a) Supercell sketches of the 12-element polarization-independent dynamic MEMS-OMS blazed gratings. Outlines of the nanobrick dimensions optimized for *T*_a_ = 20 nm and *T*_a_ = 430 nm are indicated with black squares and orange dashed lines, respectively. (b, c) Calculated diffraction efficiencies into the specular (*m* = 0) and first diffraction order (*m* = +1) as a function of wavelength with the optimal air gap for each grating, for *x*- and *y*-polarized excitations. (d, e) Calculated diffraction efficiencies as a function of air gap *T*_a_ at λ = 800 nm for *x*- and *y*-polarized excitations.

After analyzing the polarization-dependent responses of the unit cell in both GSP and FP regions, we start to implement functional metasurfaces. Figure 3 compares the performance of two blazed metasurface gratings, one optimized for *T*_a_ = 20 nm and the other for *T*_a_ = 430 nm. As illustrated in Figure 3a, the metasurface gratings consist of a periodic array of 12 elements, with the first 2 elements empty while the other dimensions are chosen and marked with blue circles in Figure 1e and Figure 1f to provide large reflection amplitudes and an approximately linear phase gradient along the *x*-direction.

In general the metasurface can be designed to control two orthogonal polarization states independently by using anisotropic elements, but the blazed grating is made polarization independent by choosing isotropic elements with *L*_*x*_ = *L*_*y*_. Figure 3b and Figure 3c show the diffraction efficiencies of the two gratings for *x*- and *y*-polarized light as a function of wavelength, where the polarization independent behavior can be observed. The efficiencies of the +1 diffraction order at the design wavelength of λ = 800 nm are similar for both gratings, with the values approaching 75%. However, the operating bandwidth is different. The grating working at *T*_a_ = 20 nm has an efficiency above 60% in the wavelength range between 760 and 900 nm, while for the grating working at *T*_a_ = 430 nm the corresponding wavelength range is only spanning from 770 to 825 nm. This reduced bandwidth is due to the fact that the FP resonance is narrower than the GSP resonance. At higher order FP resonances, the bandwidth will be reduced even further as the resonance requires the air gap to be an integer multiple of half wavelengths. Conversely, this effect might be used to design highly chromatic metasurfaces by choosing a large air gap. Figure 3d and Figure 3e compare the diffraction efficiencies of two metasurface gratings for *x*- and *y*-polarized light as a function of *T*_a_ at the design wavelength of 800 nm. Impressively, both metasurface gratings achieve more than 65% reflection in the +1 diffraction order when the air gap is *T*_a_ = 20 nm and *T*_a_ = 430 nm due to the similarity of the meta-atoms. But there is a clear gain in efficiency by tailoring the meta-atoms for the relevant air gap, which is true for both polarization states.

**Figure 4:**
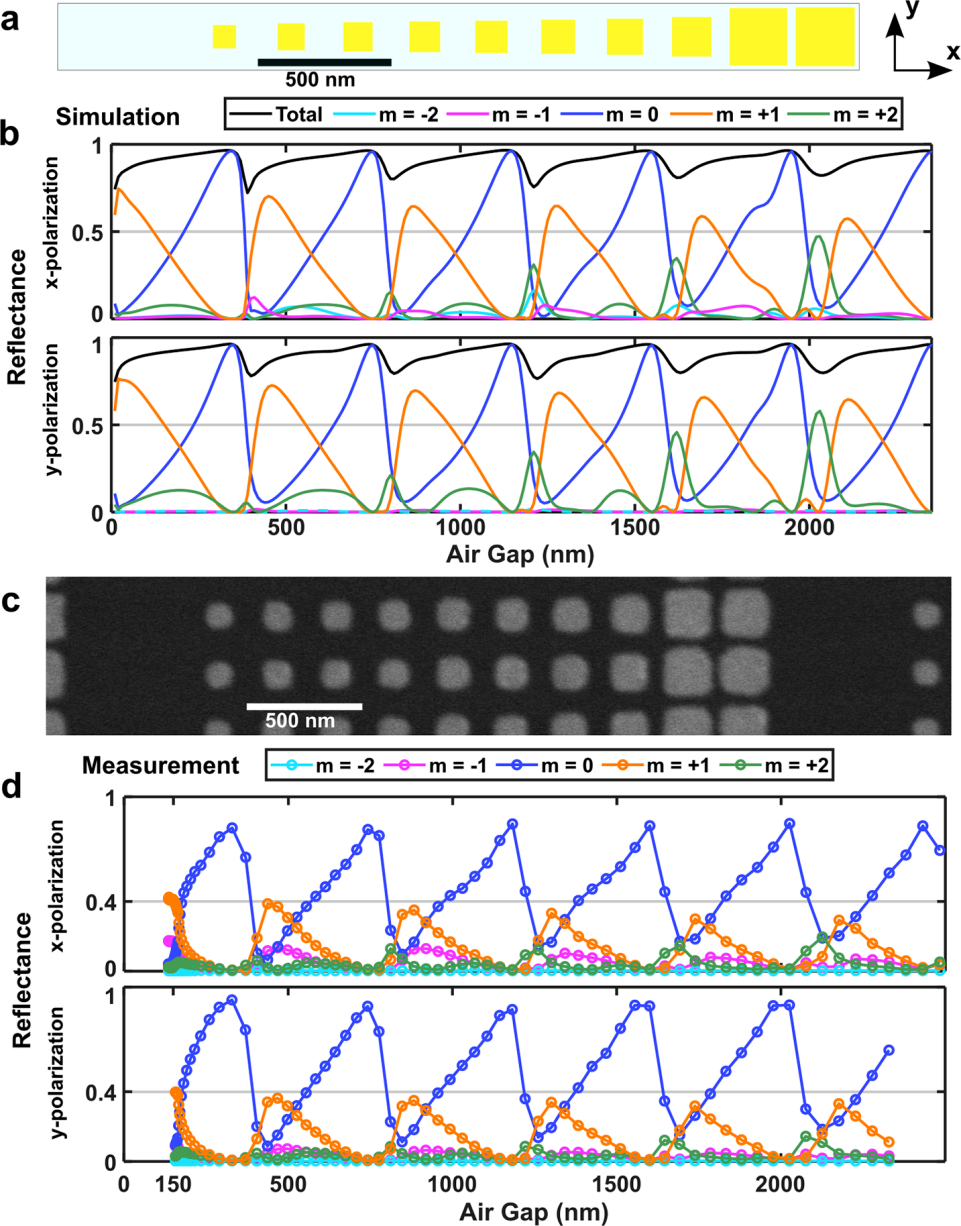
Effect of coupling via mirror substrate on grating efficiency: comparison of simulations and experimental measurements. (a) Supercell of a MEMS-OMS dynamic blazed grating with 12 meta-atoms optimized for the GSP region. (b) Diffraction efficiencies as a function of air gap *T*_a_. For these simulations the gold nanobricks are placed on a glass substrate, which is the case for the fabricated OMS shown in (c). The OMS is placed in close proximity (<3 μm) to a piezoelectric MEMS gold mirror. The air gap can then be changed by applying voltages on the MEMS. The measured diffraction efficiencies are shown in (d). The air gap values in (d) have been added by measuring the approximate relationship between air gap and voltage as described in Figure S7. In both (b) and (d) the upper (lower) plot is for *x*-polarized (*y*-polarized) excitation. The nanobrick thickness is 50 nm, and the wavelength is 800 nm.

One may expect the same responses for metasurface blazed gratings with repeating FP regions. However, in our simulations and experimental results we observe a decrease in the +1 order efficiency at higher order FP resonances in combination with more reflection into other diffraction orders, which can be understood as increased coupling or cross-talk between neighboring nanostructures via reflections in the gold substrate. Figure 4a shows another 12-element blazed grating optimized for *T*_a_ = 20 nm. As earlier, the choice of nanobrick dimensions is based on simulations where the nanobricks are placed in an array of identical neighbors, while in practical applications the neighbors may have any geometry. This has been shown to not significantly affect the performance of GSP metasurfaces as long as the phase gradient is not too large.^25^ However, as can be seen in the reflection amplitude for the diffraction orders plotted in Figure 4b, the efficiency of the grating degrades as *T*_a_ increases, with light going into unwanted diffraction modes other than the desired +1 diffraction order, falling from 72% at *T*_a_ = 430 nm to 66% at the fourth FP resonance due to increased coupling between elements within the supercell via the mirror substrate. In Figure S3, the same effect is visible for a grating made of 8 unit cells. With fewer meta-atoms the neighboring nanobricks are less similar in size, resulting in a larger drop in efficiency going from around 70% at *T*_a_ = 430 nm to less than 50% at the fourth FP resonance. The reflected field distributions for a blazed grating at several different air gap separations are shown in Figure S4. Figure 4c and Figure 4d show experimental measurements of a fabricated metasurface paired with a piezoelectric MEMS mirror, showing the same gradual decrease of the maximum diffraction efficiency from around 38% at the first FP resonance to 30% at the fourth FP resonance for both polarization states, together with increased intensity in the +2 diffraction order. This coupling issue is especially important when making high NA lenses or other components requiring large deflection angles, where the large phase gradient will require nanobricks that differ significantly from their neighbors. Details and some discussion of the fabrication and optical characterization can be found in Figures S5 and S6, while Figure S7 describes the measurements done to determine the relationship between air gap and voltage applied to the MEMS mirror. It should be noted that the minimal air gap achieved with the measured sample was ∼150 nm, sufficient for FP operation but not optimal for GSP operation. Closer separations can be achieved^23^ but likely requires significantly more effort to be produced with high yield and might be harder to realize with larger apertures. To conclude, we show how the recently developed MEMS-OMS platform is not limited to working in the GSP region. For larger air gaps the FP resonances enable the system to still function as a metasurface, with slightly smaller phase range and similar efficiencies if the nanostructure geometries are optimized to work at the relevant air gap. The main advantage of allowing for larger air gaps is alleviating issues with thin film damping for high speed operation, as well as simplifying fabrication tolerances as decreasing the air gap below 50 nm is a very challenging problem, requiring flat parallel surfaces free from any particles or irregularities that may obstruct the MEMS movement. Meanwhile, working in the GSP region gives better bandwidth and fewer issues with coupling between meta-atoms, which causes the system to change behavior between different FP periods when having metasurfaces comprised of nonidentical meta-atoms. Ultimately, the choice between two different, albeit similar, operation regimes of the considered MEMS-OMS platform should be made by carefully considering all implications of their advantages and drawbacks, highlighted in this work, to targeted functionalities and particular applications in optical systems.

## Methods

The simulations were done using COMSOL Multiphysics 5.6 with the Wave Optics module. The refractive index for gold was interpolated from experimental values^31^ for both the gold substrate and gold nanobricks. When simulating individual nanobricks, the unit cell is set to have periodic conditions in both *x*- and *y*-directions, while the gold substrate is backed by a perfect electrical conductor condition and the air region is padded with a perfectly matched layer. When using eq 1 the coefficients *r*_12_, *r*_21_, *t*_12_, and *t*_21_ are determined by simulating the nanobricks without the gold substrate, with perfectly matched layers backing the domains on both sides of the nanostructure in the *z*-direction. *r*_23_, the reflection coefficient for the gold substrate for light with normal incidence, is determined by
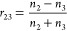
*n*_2_ being the refractive index of the layer above the substrate (in this case air, *n*_2_ = 1) and *n*_3_ the refractive index of gold. The light was set to be normally incident for all simulations, and the results were calculated independently for light linearly polarized in the *x*- and *y*-directions. When simulating gratings with large air gaps and many nanobricks, the simulation volume was reduced by simulating half the unit cell and replacing the periodic boundary conditions in the *y*-direction by perfectly magnetic or electric conductors depending on the incident polarization state. For the simulations in Figure 4, the nanobricks were placed on a lossless dielectric material with refractive index 1.46.

Fabrication of the MEMS metasurface devices is done by individually manufacturing MEMS micromirrors and glass substrates containing the metasurfaces, before manually gluing the MEMS chips and glass substrates together in a cleanroom environment. A description and discussion of this process can be found in Figure S5. Additional details can also be found in refs (24) and (32) for MEMS fabrication and refs (23) and (28) for the MEMS–metasurface combination.

Optical characterization is done by sending laser light with wavelength 800 nm through a linear polarizer, half wave plate (used to switch between two linear polarization states), and beam splitter and then focusing onto the sample using a microscope objective. The light is reflected back into the objective and is redirected by the beam splitter, before tube lens, iris (in image plane for spatial filtering), and two lenses that relay the light onto a CMOS camera. The last lens can be flipped in and out of the optical path to switch between capturing the direct and Fourier images. The direct object image is used to ensure the signal is collected from only the metasurface area, while the intensity in the different diffraction orders is measured by integrating the intensity of the corresponding areas in the Fourier plane image. Details on the equipment and a diagram of the setup can be found in Figure S6.

## Supporting Information Available

Figure S1 showing phase and amplitude profiles for the blazed metagratings; Figure S2 showing error of eq 1; Figure S3 showing simulation results for a metasurface blazed grating with 8 elements; Figure S4 showing plots of reflected field distribution for a blazed grating at several air gap values; Figure S5 showing details of the fabrication of the MEMS metasurface devices with relevant discussion; Figure S6 showing a schematic of optical measurement setup; Figure S7 showing characterization and discussion of air gap as a function of voltage (PDF)
