# Supplementary material for: MEMS Tunable Metasurfaces Based on Gap Plasmon or Fabry–Pérot Resonances
Source: Nano Lett. 2022 Aug 18;22(17):6951–7. doi: 10.1021/acs.nanolett.2c01692 (PMC9479152; doi:10.1021/acs.nanolett.2c01692)
Supplement: Supplementary file 1 — nl2c01692_si_001.pdf [file nl2c01692_si_001.pdf]

MEMS Tunable Metasurfaces Based on Gap Plasmon or Fabry-Pérot Resonances

Supplementary Information

Paul C. V. Thrane[1,2,3], Chao Meng[1,3], Fei Ding[1], Sergey I. Bozhevolnyi[1]*

[1] Centre for Nano Optics, University of Southern Denmark, Campusvej 55, Odense DK-5230, Denmark.

[2] SINTEF Smart Sensors and Microsystems, Gaustadalleen 23C, 0737 Oslo, Norway.

[3] These authors contributed equally to this work.

* seib@mci.sdu.dk

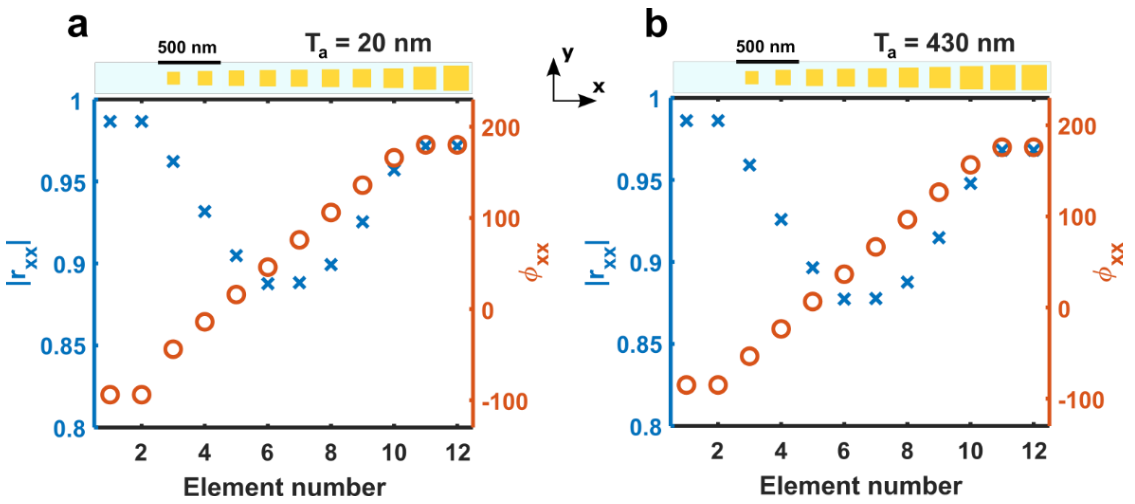

Figure S1. Reflection amplitudes (crosses) and phases (circles) for the nanobrick geometries chosen for polarization-independent beam steering metasurfaces optimized for T_{a} = 20 nm (a) and T_{a} = 430 nm (b). The values are calculated for normally incident x-polarized excitation at the wavelength of λ = 800 nm, The choice of nanobricks is also indicated with circles in Figure 1e and 1f. The supercell consisting of 12 meta-atoms (where 2 have been left without nanobricks, which is done mainly to simplify fabrication) is shown above each plot. The simulated efficiency and bandwidth of these gratings is presented in Figure 3.

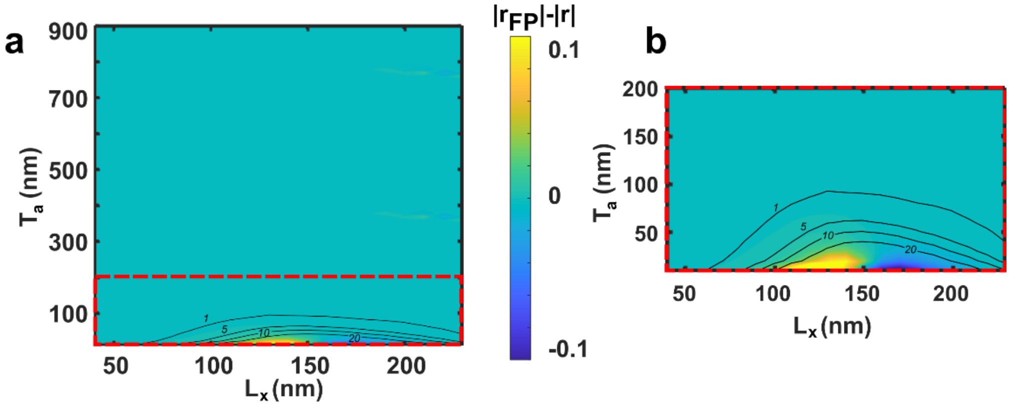

Figure S2. (a) Deviation between the reflection coefficient (|r|) found through simulating the full system of bricks including gold substrate, and the reflection coefficient ($|r_{\mathrm{FP}}|$) calculated with Equation 1 using simulations of bricks without any substrate. The color map shows the error of the reflection amplitude, while the black contour lines show phase error with numbers in degrees. (b) Close up showing the region where the FP equation does not give correct results because of near field coupling between the nanobricks and gold substrate around the GSP resonance. For these simulations all the bricks are identical and have a square footprint with $L_y = L_x$, $\lambda = 800$ nm, $\Lambda = 250$ nm and $t_m = 50$ nm.

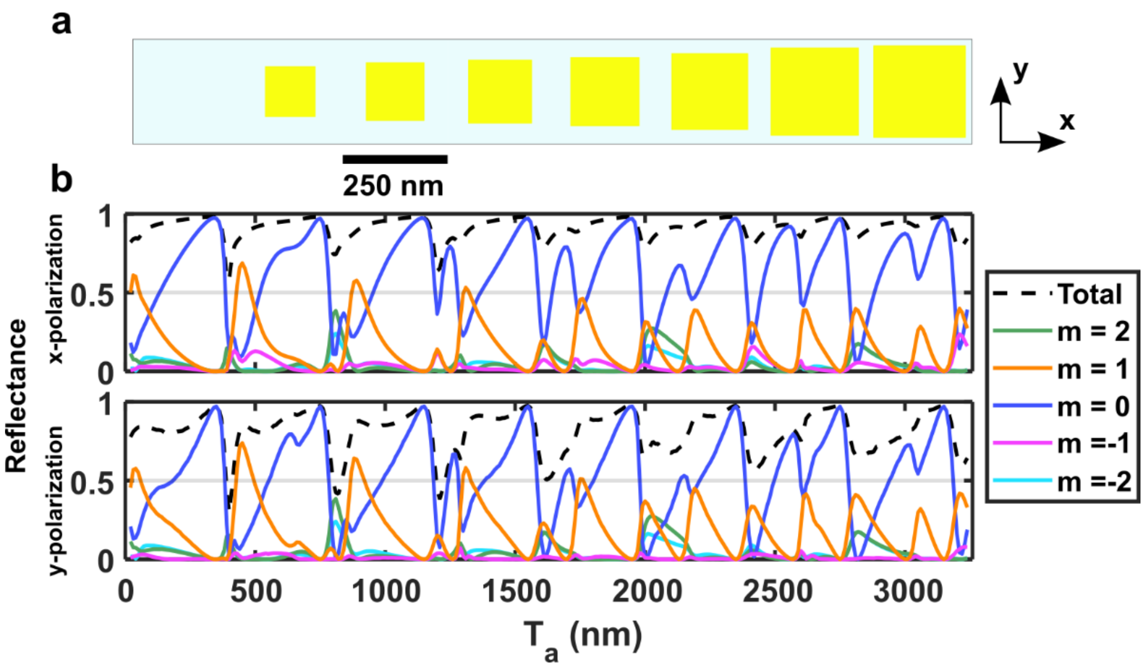

Figure S3. Intercell coupling of MEMS-OMS in the FP regime. (a) Supercell sketch of an 8-element MEMS-OMS blazed grating, optimized for $T_{\mathrm{a}} = 430$ nm. (b) Simulated diffraction efficiencies (m≤2) as a function of the air gap T_{a} for normally incident *x*- (upper) and *y*-polarized (lower) light, $\lambda = 800$ nm.

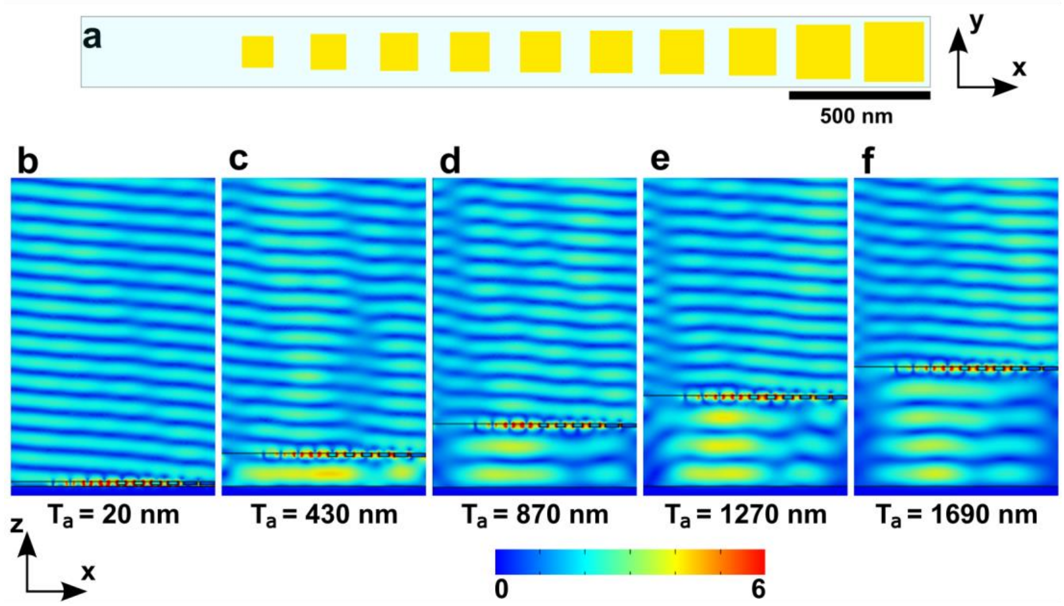

Figure S4. Field distributions from a blazed grating for several air gaps corresponding to GSP mode (a), and the first four FP modes (b-e). The grating is optimized for GSP operation and is the same as is shown in Figure 3. The plotted intensity is for light linearly polarized along the x-axis. Reflection into the first diffraction order is better for smaller separations, with more unintended scattering happening for higher FP modes.

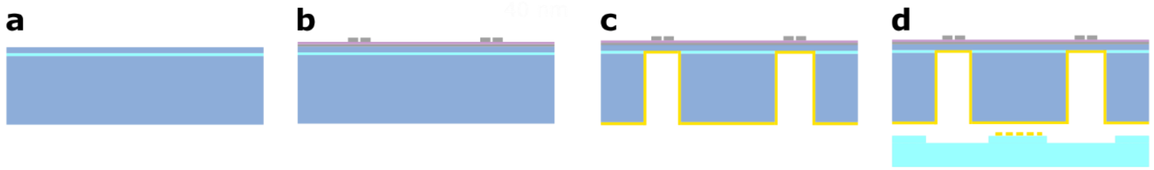

Figure S5. Overview and discussion of the fabrication process. The subfigures illustrate a cross-section through the center of the MEMS chip in four stages of the fabrication process. More details of the MEMS micromirror fabrication is found in [24,32], a brief summary is as follows: Starting from a silicon on insulator wafer (a), we deposit a bottom electrode of platinum, thin-film layer of the piezoelectric material lead zirconate titanate (PZT) using chemical solution deposition and finally top electrodes of gold (b). An annulus trench is etched from the back side of the wafer with deep reactive ion etching, leaving a central silicon mass suspended by a PZT membrane. The back side of the wafer is subsequently covered in gold to make the silicon mass function as a mirror (c). By applying voltages to the top electrodes, the PZT membrane can be deformed thus allowing the mirror in the center to be moved. This MEMS device is then glued to a glass substrate (Loctite 401 Cyanoacrylate glue) with a plasmonic metasurface (d). To make the metasurface, the glass substrate (Borofloat 33, Wafer Universe) is first etched in an annulus area to reduce the probability of particle contamination impeding movement of the micromirror. Then, the gold nanostructures are fabricated on top of the resulting pedestal using electron-beam lithography and lift-off technique: Deposition of 100 nm PMMA A2 from MicroChem, 40 nm conductive polymer AR-PC 5090 from Allresist. Definition of the nanostructure pattern using JEOL JSM-6500F field-emission SEM with a Raith Elphy Quantum lithography system, then deposition of 1 nm titanium followed by 50 nm gold and finally lift-off in acetone. Fabrication of similar MEMS-metasurface devices has also been described in [23,28]. The MEMS were made using batch processing on 6-inch wafers, while the metasurfaces were done one chip at a time. Typically, the separation distance after gluing MEMS and glass substrate together is found to be around 3 µm, well within the micromirror range of +/- 6 µm. Most of the fabricated devices reach a closest separation of approximately 150-400 nm, which allows for operation in the FP regime. GSP operation for the wavelengths discussed here (around 800 nm) requires sub 100 nm separation which has been demonstrated [23] but likely requires heterogeneous integration of the fabrication processes in a clean-room environment to have significant yield, in our case less than 10 % of fabricated samples came close to these separation distances. Switching speed is highly dependent on the resonance frequency which is decided by MEMS characteristics such as mirror mass and membrane stiffness, the MEMS devices used in this work were optimized for mirror flatness, long movement range and low operation voltage, not for speed, but found to have switching speeds around 0.3 ms [28].

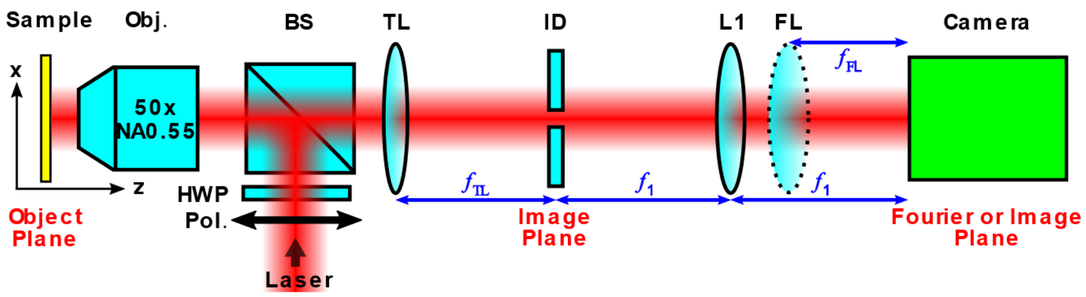

Figure S6. Experimental setup for MEMS-OMS characterization. Sample: MEMS-OMS component; Laser: Tunable CW Ti:sapphire laser (Spectra-Physics 3900S, wavelength range: 700-1000 nm); Pol.: Polarizer (LPNIR050-MP2, Thorlabs); HWP: Half-wave plate (AHWP10M-980, 690-1200 nm, Thorlabs); BS: Beam splitter (CCM1-BS014, 700-1000 nm, Thorlabs); Obj.: Objective (M Plan Apo 50×/0.55, Mitutoyo); TL: Tube lens (TTL200-S8, Thorlabs); ID: Iris (ID12Z, Thorlabs); L1: Lens (AC-254-125-B-ML, f = 125 mm, Thorlabs); FL: Flip lens (AC-254-100-B-ML, f = 100 mm, Thorlabs); Camera: CMOS Camera (DCC1545M-GL, Thorlabs). ID is located at the first image plane for filtering out the area of interest in the measurement. The FL is used to switch from Fourier image to direct image.

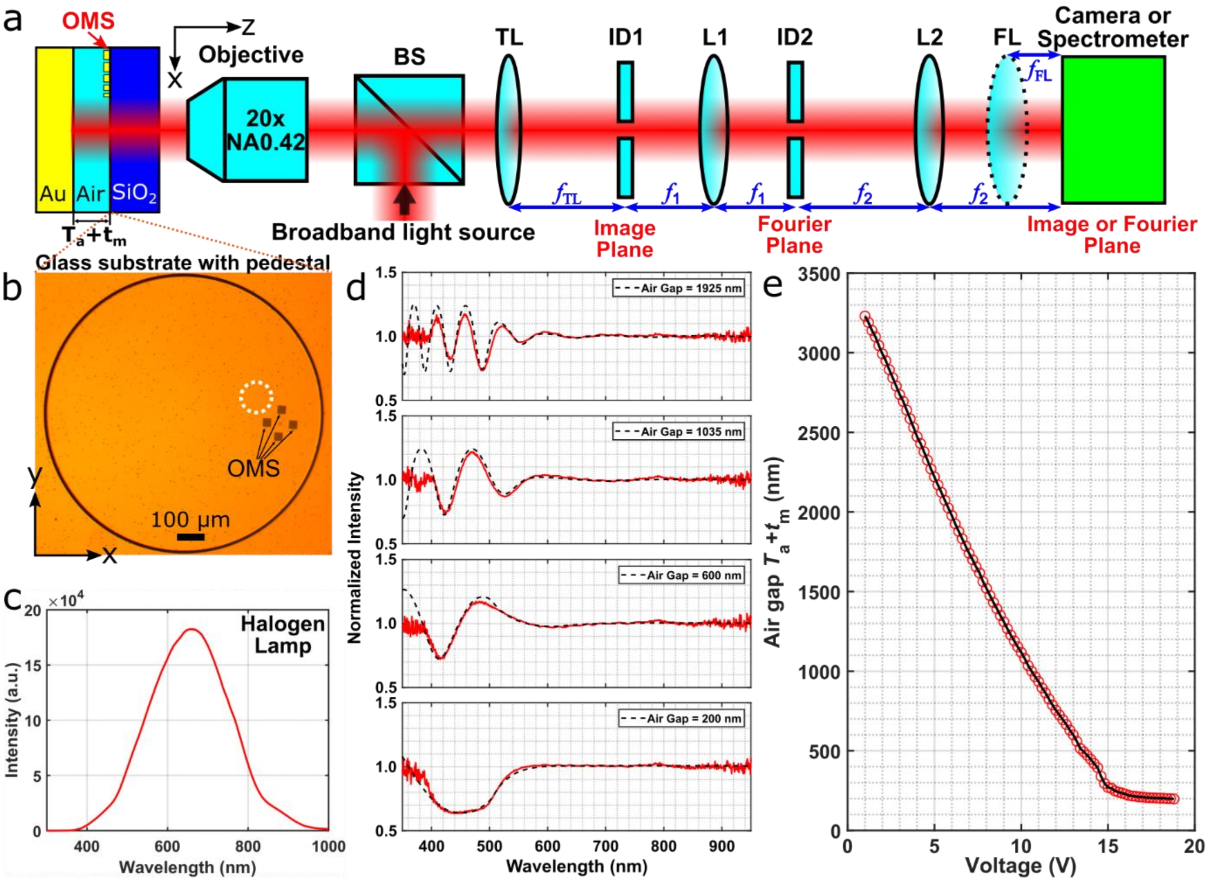

Figure S7. Characterization of air gap as a function of voltage applied to MEMS mirror. When actuating the thin-film PZT piezoelectric MEMS mirrors there is some amount of hysteresis and nonlinearity. Therefore, in practical applications where one requires single nm-level positioning it is necessary to have some sort of feedback mechanism, for example optical, piezoresistive or capacitive. The sample measured for this work did not have any such feedback system, and thus measurement of the air gap as a function of voltage was conducted separately, as described in this figure and caption, allowing

determination of the closest separation which was found to be around 200 nm, corresponding to a nanostructure-mirror separation of 150 nm. The measurement was done by looking at the reflection spectra of broadband light incident on the glass substrate and mirror in an area beside the metasurface, thus not being affected by the metasurface but still measuring the air gap in the relevant area of the device. (a) Experimental setup for estimating air gaps between OMS and MEMS mirror. Broadband light source: Halogen lamp (KL1500 LCD, Schott, wavelength range: ~400-1000 nm); BS: Beam splitter (CCM1-BS013, 400-700 nm, Thorlabs); Obj.: Objective (M Plan Apo 20×/0.42, Optem); TL: Tube lens (TTL200-A, Thorlabs); ID1: Iris 1 (SM1D12SZ, Thorlabs); L1: Lens 1 (AC-254-150-A-ML, f = 150 mm, Thorlabs); ID2: Iris 2 (ID25, Thorlabs); L2: Lens 2 (LB1904-A-ML, f = 125 mm, Thorlabs); FL: Flip lens (LBF254-100-A, f = 100 mm, Thorlabs); Camera: CMOS camera (DCC1645C-HQ, Thorlabs); Spectrometer: VIS-NIR spectrometer (QE pro, Ocean Optics). ID1 and ID2 are located at the first image and Fourier plane for filtering out the area of interest in the measurement. The FL is used to switch from direct image to Fourier image. (b) Optical microscopy image of the OMS fabricated atop a 10-µm-high pedestal on the glass substrate and used for the measurement in Figures 4 and S7. The dashed circle marked the area that is right next to the OMS and used for estimating the air gap sizes with actuated voltages. (c) Measured spectrum of the incident broadband light from a halogen lamp. (d) Typical measured spectra of the reflected light for the glass (1-mm-thick)/air/gold (100-nm-thick) structure, normalized by the reflected light of a glass (1-mm-thick)/gold (100-nm-thick) structure. A simple Fabry–Pérot etalon model is used for fitting the measured results (solid red lines). The dashed line is the fitting results with the analytical method, obtained with different air gap sizes of 1925, 1035, 600 and 200 nm, respectively. (e) Estimated air gap sizes ($T_a + t_m$) as a function of the applied voltages.
